# Impact of three years of large scale Indoor Residual Spraying (IRS) and Insecticide Treated Nets (ITNs) interventions on insecticide resistance in *Anopheles gambiae s.l*. in Benin

**DOI:** 10.1186/1756-3305-5-72

**Published:** 2012-04-10

**Authors:** Gil Germain Padonou, Michel Sezonlin, Razaki Ossé, Nazaire Aizoun, Frédéric Oké-Agbo, Olivier Oussou, Ghélus Gbédjissi, Martin Akogbéto

**Affiliations:** 1 Faculté des Sciences et Techniques de l'Université d'Abomey Calavi, Calavi, Bénin; 2 Centre de Recherche Entomologique de Cotonou (CREC), Cotonou, Bénin

**Keywords:** IRS, LLIN, Bendiocarb, Deltamethrin, Resistance, *Kdr*, *Ace-1^R^*, *Anopheles gambiae*, Benin

## Abstract

**Background:**

In Benin, Indoor Residual Spraying (IRS) and long-lasting insecticidal nets (LLINs) are the cornerstones of malaria prevention. In the context of high resistance of *Anopheles gambiae *to pyrethroids, The National Malaria Control Program (NMCP) has undertaken a full coverage of IRS in a no-flood zone in the Oueme region, coupled with the distribution of LLINs in a flood zone. We assessed the impact of this campaign on phenotypic resistance, *kdr *(knock-down resistance) and *ace-1^R ^*(insensitive acetylcholinesterase) mutations.

**Methods:**

Insecticides used for malaria vector control interventions were bendiocarb WP (0.4 g/m^2^) and deltamethrin (55 mg/m^2^), respectively for IRS and LLINs. Susceptibility status of *An. gambiae *was assessed using World Health Organization bioassay tests to DDT, permethrin, deltamethrin and bendiocarb in the Oueme region before intervention (2007) and after interventions in 2008 and 2010. *An. gambiae *specimens were screened for identification of species, molecular M and S forms and for the detection of the West African *kdr *(L1014F) as well as *ace-1^R ^*mutations using PCR techniques.

**Results:**

The univariate logistic regression performed showed that *kdr *frequency has increased significantly during the three years in the intervention area and in the control area. Several factors (LLINs, IRS, mosquito coils, aerosols, use of pesticides for crop protection) could explain the selection of individual resistant *An. gambiae*. The *Kdr *resistance gene could not be the only mechanism of resistance observed in the Oueme region. The high susceptibility to bendiocarb is in agreement with a previous study conducted in Benin. However, the occurrence of *ace-1^R ^*heterozygous individuals even on sites far from IRS areas, suggests other factors may contribute to the selection of resistance other than those exerted by the vector control program.

**Conclusion:**

The results of this study have confirmed that *An.gambiae *have maintained and developed the resistance to pyrethroids, but are still susceptible to bendiocarb. Our data clearly shows that selection of resistant individuals was caused by other insecticides than those used by the IRS and LLINs.

## Background

*Anopheles gambiae *Giles (Diptera: Culicidae) is the major malaria vector in West Africa. In Benin it mainly transmits *Plasmodium falciparum *which is responsible for malaria [1]. *An.gambiae *exists in two distinct molecular forms, referred to as 'M' and 'S' based on the variation observed in molecular markers [2]. In sub-Saharan Africa, insecticide treated nets (ITNs) and indoor residual insecticide spraying (IRS) are the cornerstones of malaria vector control [3]. These vector control methods aim to reduce morbidity and mortality caused by malaria. ITNs and IRS have each been shown to be highly effective methods of malaria vector control in their own right. A recent review of the evidence of cost and consequences of large-scale vector control for malaria concluded that both ITNs and IRS are highly cost effective vector control strategies [4]. ITNs have been the mainstay of vector control in many countries in which the disease is endemic and where infrastructure limits or precludes the implementation of IRS [5]. Unfortunately the resistance of *An.gambiae *to insecticides used for malaria vector control has occurred. This resistance has been associated with all insecticidal compounds used for insect vectors of human disease, including African malaria vectors [6]. The ongoing spread of insecticide-resistant genes, such as the well-characterized *kdr *mutations [7,8] in populations of the major African malaria vectors, *An. gambiae *and its sibling species *An. arabiensis *[9-12], can seriously jeopardize the efficacy of vector control programs [13]. It has been shown that in West and West-Central Africa, the L1014F allele was frequent in the S molecular form of *An. gambiae *[9,14,15], whereas only few M form populations from the gulf of Guinea presented kdr-w alleles at low frequencies [14,15], except in a few urban and peri-urban coastal areas where it reached high frequencies [16,17]. Several recent studies conducted in Benin [18-21] have also indicated that *An.gambiae *is highly resistant to pyrethroids and DDT, but not to bendiocarb. It is in this context that the National Malaria Control Program (NMCP) has undertaken a full coverage of the IRS in no-flood zones in the Oueme region coupled with the distribution of mosquito treated nets in flood zones. In the situation of vector resistance to pyrethroids, the ability to use other families of insecticides is one of the alternatives available for the malaria vector control. Thus, bendiocarb WP, which gave good results in experimental huts [20], was chosen by the NMCP for the IRS at the community level. Following the first spraying campaign implemented (July/August 2008), three other cycles (March/April 2009, March/April 2010, July/August 2010) of treatment were conducted in the Oueme region. Despite the residual activity of bendiocarb which was 4 months on cement surfaces [20], the number of rounds in 2008 and 2009 was dependent on financial resources available. IRS was not implemented in the flood zone because of the presence of water bodies, which could be at risk of contamination by insecticides. Therefore, 48,819 LLINs (Long-Lasting Insecticidal Nets, Permanet 2.0) were distributed to 47,524 households, with particular attention to children under-five and pregnant women, in October 2008 and May 2009. A quantity of 35,120 kg of deltamethrin 100% (719.4 mg per net) was contained in 48,819 LLINs distributed in the flood zone. For house spraying, a total of 128,132 kg of bendiocarb 80% was sprayed onto the walls of 166,910 human dwellings to protect a population of 512,491 in a no-flood zone.

Under these conditions, it is possible that the level of initial resistance has changed. Elsewhere in East Africa, no selection effect from the long-term use of ITNs on phenotypic resistance was noticed [22,23], whereas other studies reported a rapid increase of *kdr *mutation after four years of ITNs community use in Kenya [24] and in Equatorial Guinea [17] following a large-scale insecticide residual spraying (IRS) program. There was a similar case in West Africa, where an increasing Leu-Phe knockdown resistance mutation in *Anopheles gambiae *from Niger following a nationwide long-lasting insecticide-treated nets implementation at the end of 2005 [25] was reported. Other studies have shown the effect of insecticide treated nets (ITNs) with pyrethroids on *An. gambiae *populations and the possible selection of *kdr *alleles either in laboratory experiments [26] or experimental huts trials [27]. In Benin, the susceptibility levels of populations of *An. gambiae *to carbamates and organophosphates, the association of the reported high *kdr *frequency with the resistance phenotype, and the occurrence of other possible mechanisms of resistance are poorly understood. The present study aimed to report the first case of the impact of the three years of large scale of IRS and LLINs interventions on phenotypic resistance, *kdr *and *ace-1^R ^*alleles in natural populations of *An. gambiae s.l*. from southeast Benin. The results provide crucial information about potential effects of wide-scale IRS and LLIN coverage on *kdr *and *ace-1^R ^*mutation selection and possible effects on phenotypic resistance to deltamethrin and bendiocarb in order to improve the malaria vector control programs.

## Methods

### Study area

**Figure 1:**
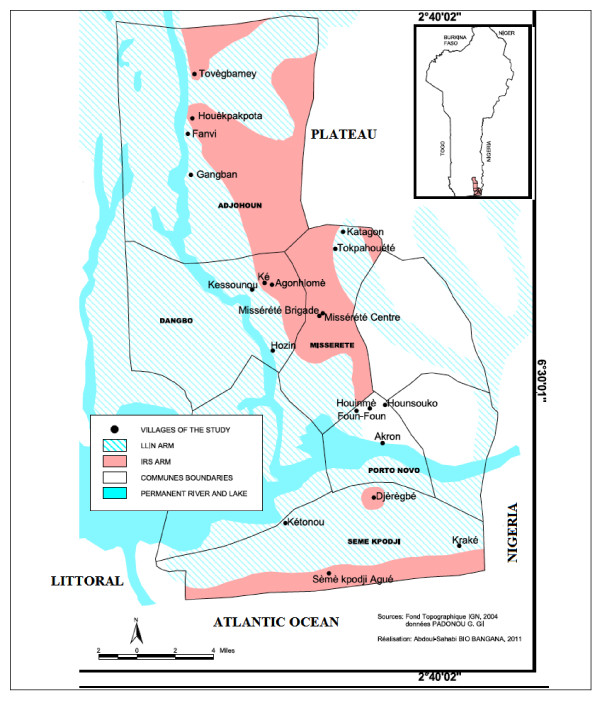
**Map of the study area**.

The study area is located in the Southeast of Benin (West Africa) and includes four districts of the Oueme region: Adjohoun, Dangbo, Misserete and Seme (Figure 1). The four districts covered 977 km^2 ^and an estimated 64,799 households. Oueme region has a sub-equatorial type climate with a monthly average temperature ranging from 20°C to 34°C and an annual average rainfall between 1,300 and 1,700 mm. Malaria transmission is stable in the Oueme region, which is irrigated by the river Oueme, Lake Nokoue and the lagoon of Porto-Novo. These streams determine two ecological zones in the Oueme region: a plateau zone and a flood zone. In the present study, the plateau zone is referred to as the" IRS area", and the flood zone is called "LLIN area". An estimated distance between 5 and 7 km separated the plateau and the flood areas. This distance was sufficient enough to prevent migration of mosquitoes from one area to another. The density of the human population was as high in IRS areas as in the flood zone, so that mosquitoes do not need to fly far away for blood feeding [28]. According to RTI, the coverage rate of IRS was more than 90% for each of the first three rounds.

In a context where universal access to LLINs was promoted, it was not easy to find a good control area. However, Porto-Novo, an area that presents the same ecological and geographical characteristics as the four districts mentioned above was chosen as control.

There is no IRS and free distribution of LLINs in Porto-Novo. Nevertheless some people who had bed nets, especially children and pregnant women, used them, but the proportion of consistent users was low. Before IRS implementation and the free distribution of LLINs, a baseline study of phenotype resistance with *kdr *and *ace-1^R ^*frequencies in *An.gambiae *populations was carried out in the study area. The baseline data is shown here for a comparison before and after interventions.

### Insecticides used for IRS and ITNs

Bendiocarb 80%WP (Wettable powder) was selected for spraying onto the walls in IRS area. The application dose was 0.4 g/m^2 ^of bendiocarb on walls of houses. The four applications were implemented by volunteers selected from the local community and trained by the Research Triangle Institute (RTI) team, the implementing partner of the U.S. Agency for International Development. Nets distributed in the flood zones (LLIN area) were PermaNet 2.0. PermaNet 2.0 is a WHO recommended polyester LLIN coated with the pyrethroid deltamethrin to a target dose of 55 mg/m^2 ^(± 25%).

### Study design and mosquito collections

The mosquito sampling was conducted before the implementation of the IRS and LLIN free distribution, to provide baseline data on *kdr *and *ace-1^R ^*mutation frequency, whereas other collections were carried out during two years after interventions (in 2009, 13 months after the first round of IRS and 11 months after the first LLIN distribution; in 2010, 24 months after the first round of IRS and 23 months after the first LLIN distribution). To carry out this sampling, four villages, including two in the IRS area and two in the LLIN area were randomly selected in each district and two human dwellings were chosen per village for mosquito collection using human landing catches (HLC). Similarly, four villages were chosen in the control area that had received no intervention (two as IRS control area and two as LLIN control area). Adult mosquitoes were collected twice a month with one collector located inside and another outside in each village. Mosquito collections were carried out twice a month, during three months in the wet season (September to November) in 2007, 2009 and 2010. The same human dwellings were used for HLC during the study and their characteristics were the same throughout the study. Female *An. gambiae *species were morphologically identified using morphological keys [29] and put into microtubes with dessicant, and then stored between -20 and -28°C in the laboratory before processing. Additionally in the same period of wet season in 2007, 2009 and 2010, some larval samples were simultaneously collected. Ten of the previous villages including two in each district of Dangbo and Misserete IRS area, two in each district of Adjohoun and Seme LLIN area, two in the control area were taken into account. In each village selected *An. gambiae *larvae and pupae were collected using the dipping method on several breeding sites (brick pits, pools, marshes, streams, ditches, pits dug for plastering traditional huts, puddles of water, water pockets caused by the passage of cattle and gutters) near human dwellings where the conditions of blood meals are available for Anopheles. The larvae and pupae were kept in separated labeled bottles related to each locality. Some of the larval samples were reared up to adult emergence at the CREC (Centre de Recherche Entomologique de Cotonou, Benin) insectary under standard conditions (25 ± 2°C; 80% ± 4%: Relative Humidity), for further bioassay tests. A strain of *An. gambiae *(Kisumu) was used as reference strain to compare the susceptibility levels of the field populations.

### Species identification

All mosquitoes collected by HLC and all live and dead specimens of *An. gambiae *from the bioassay test were subjected to the *An. gambiae *species specific PCR assays for species identification [30]. Aliquots of DNA extracted from PCR positive specimens of *An. gambiae *s.s. were subjected to PCR assays for identification of the molecular 'M' and 'S' forms [31].

### PCR detection of the *kdr *and *ace.1^R ^*mutations

Polymerase chain reaction diagnostic test for detection of *kdr *"Leu-phe" mutation was carried out on *An. gambiae *mosquitoes as described by Martinez-Torres *et al. *[7]. The PCR-RFLP diagnostic test was used to detect the presence of G119S mutation (*ace.1^R ^*gene) as described by Weill *et al. *[32].

### Insecticide susceptibility test

The insecticide susceptibility test was carried out before and after the interventions in two districts (Dangbo and Misserete) of the IRS area and in two others districts (Adjohoun and Seme) for the LLIN area. Female mosquitoes aged 2-5 days old were exposed to diagnostic doses of various insecticides for susceptibility tests using insecticide-impregnated papers, as described by the standard WHO testing protocol [26]. The following insecticides were tested: deltamethrin (0.05%), permethrin (0.75%), DDT (4%) and bendiocarb (0.1%). The emphasis was also put on deltamethrin, because of a distribution of PermaNets by the NMCP in the swampy area. The use of DDT is justified by the detection of cross-resistance between pyrethroids and organo-chlorine in *Anopheles *populations [9]. Bendiocarb (carbamate) was the insecticide used in the IRS area situated far from flood zone. For each district, five test tubes were used: one untreated paper as a control and four treated papers to expose mosquitoes. Control tubes contained filter papers impregnated with silicon oil (insecticide carrier) only, whereas treated papers were impregnated with diagnostic doses of insecticide plus carrier. An average of twenty-five mosquitoes were introduced into each tube. Females of *An. gambiae *used in this study were exposed for one hour to insecticide-treated papers and monitored at different time intervals (10, 15, 20, 30, 45, 60 minutes) to record the "knock-down" times. After 1 hour exposure, mosquitoes were transferred into holding tubes and provided with cotton wool wet with a 10% honey solution. Mortalities were recorded after 24 hours and the susceptibility status of the population was graded according to the WHO recommended protocol [33]. Dead and surviving mosquitoes from this bioassay were kept separately in Carnoy solution at -20°C for further molecular characterization.

### Statistical analysis

Using R software version 2.11.1 [34], a univariate logistic regression, was performed with *kdr *frequency as the dependent variable and the year as a covariate with ANOVA test to determine the association of *kdr *frequency (dependent variable) on the one hand with the localities and also with the years 2007, 2009 and 2010 (covariates) on the other hand. This regression has also been used to appreciate the *kdr *frequency in the intervention areas compared to the control areas. This was the same to test the association between mortality rates (dependent variable) to insecticides and localities (covariates). The ANOVA test was used to assess this association. The Wald test has been used to compare *kdr *frequency and mortality rates in the intervention areas with the control areas. To compare the *ace-1 *frequency between the intervention areas and the control areas we used the Fisher exact test (Genepop software) [35] as the gene is rarely observed in mosquitoes tested. Similarly the comparison of the *kdr *and *ace-1 *frequencies from one year to another in each locality was performed using Fisher's exact test and chi-square test. A Kendall correlation test was used to study the correlation between mortality rates and survivors to deltamethrin with *kdr *frequency. The significance level was set at 5%.

### Ethical approval

This study received the approval of the Ministry of Health and the National Ethics Committee. The voluntary mosquito collectors gave their consent before participating in the study. They were also subjected to regular medical check-ups with preventive treatments of malaria. They were all vaccinated against yellow fever.

## Results

### Species and molecular forms of *Anopheles gambiae*

Species and molecular forms of *An. gambiae *s.l. collected from 2007-10 by HLC are shown in Table 1. During this study, *An gambiae s.s *was the only member identified in the *An. gambiae *complex. The analysis showed that all *An. gambiae s.s *collected were molecular M form. No S form was found during the study period.

**Table 1:** Species identification, molecular forms, *kdr *and *ace-1^R^*frequencies in *An.gambiae s.l*. collected by HLC

			Species	Molecular forms	*kdr *mutation (M form)				*ace.1^R ^*mutation (M form)			
**Localities**	**Years**	**N**	**Ag**	**M form**	**RR**	**RS**	**SS**	***kdr *f**	**RR**	**RS**	**SS**	***ace.1^R ^*f**

Control IRS	2007	22	22	22	20	2	0	0.95^a^	0	0	22	0^a^

	2009	21	21	21	18	3	0	0.92^a^	0	0	21	0^a^

	2010	39	39	39	35	04	0	0.95^a^	0	5	34	0.06^a^

Adjohoun IRS	2007	74	74	74	28	34	12	0.61^a^	0	0	74	0^a^

	2009	122	122	122	48	56	18	0.62^a^	0	0	122	0^a^

	2010	24	24	24	21	3	0	0.94^b^	0	0	24	0^a^

Dangbo IRS	2007	150	150	150	85	59	6	0.76^a^	0	0	150	0^a^

	2009	263	263	263	133	115	15	0.72^a^	0	0	263	0^a^

	2010	68	68	68	65	3	0	0.98^b^	0	4	64	0.03^b^

Misserete IRS	2007	89	89	89	58	31	0	0.83^a^	0	0	89	0^a^

	2009	129	129	129	86	35	8	0.80^a^	0	0	129	0^a^

	2010	46	46	46	43	3	0	0.97^b^	0	2	44	0.02^b^

Sèmè IRS	2007	122	122	122	109	1	12	0.90^a^	0	0	122	0^a^

	2009	190	190	190	143	17	30	0.80^b^	0	0	190	0^a^

	2010	22	22	22	19	3	0	0.93^a^	0	6	16	0.13^b^

Control LLIN	2007	150	150	150	110	40	0	0.87^a^	0	0	150	0^a^

	2009	101	101	101	90	11	0	0.95^b^	0	0	101	0^a^

	2010	43	43	43	38	5	0	0.94^b^	0	0	43	0^a^

Adjohoun LLIN	2007	52	52	52	14	30	8	0.56^a^	0	0	52	0^a^

	2009	24	24	24	8	16	0	0.67^a^	0	0	24	0^a^

	2010	17	17	17	15	2	0	0.94^b^	0	0	17	0^a^

Dangbo LLIN	2007	124	124	124	66	54	4	0.75^a^	0	0	124	0^a^

	2009	96	96	96	50	40	6	0.73^a^	0	0	96	0^a^

	2010	58	58	58	55	3	0	0.97^b^	0	3	55	0.02^a^

Sèmè LLIN	2007	72	72	72	60	0	12	0.83^a^	0	0	72	0^a^

	2009	44	44	44	31	12	1	0.84^a^	0	0	44	0^a^

	2010	7	7	7	7	0	0	1^a^	0	0	7	0^a^

Ag: *An. gambiae*; *kdr *f: *kdr *frequency; *ace.1 ^R ^*f: *ace.1 ^R ^*frequency. Numbers in the same column sharing the same superscript do not differ significantly (p > 0, 05)

### Kdr and *ace.1^R ^*frequencies in *An. gambiae s.l*. collected by HLC

The *kdr *mutation was the main mechanism of pyrethroid resistance identified in all localities from 2007 to 2010. Univariate logistic regression, performed with *kdr *frequency as the dependent variable and year as a covariate with ANOVA test, showed for the whole IRS area, that the *kdr *frequency was associated with the time (p < 0.05) and decreased significantly in 2009 compared to 2007 (OR = 0.756 < 1; p < 0.05). However, it has increased significantly in 2010 compared to 2009 (OR = 8.120 > 1; p < 0.05). Conversely, in the LLIN area, the increase in *kdr *frequency was not significant in 2009 compared to 2007 (OR = 1.295 > 1; p > 0.05) but it was significant in 2010 compared to 2009 (OR = 5.107 > 1; p < 0.05). Indeed, the *kdr *gene frequencies observed were similar in 2007 and 2009 in Dangbo, Misserete, Adjohoun and Seme LLIN area but had significantly increased in 2010 (Table 1). In the control area the level of *kdr *gene frequencies was very high and stable from 2007 to 2010 (p > 0.05). The *ace-1^R ^*mutation was 0% from 2007 to 2009 in all localities. But in 2010, heterozygous (RS) individuals of *ace-1^R ^*mutation were detected in all localities 24 months after the first round of IRS, except in Adjohoun, Seme LLIN and control LLIN. This variation (0-13%) was significant in Seme IRS in contrast to insignificant increase (p > 0.05) noticed in the localities of Control IRS, Dangbo IRS, Misserete IRS and Dangbo LLIN. A univariate logistic regression with ANOVA test showed that the increase of *kdr *frequencies is associated with the intervention areas and similarly with the control area (p < 0.05). As the *ace-1^R ^*allele was rarely observed in mosquitoes tested, the Fisher exact test, revealed that the *ace-1^R ^*frequency was similar in the intervention communities compared to the control area (p > 0.05).

### Insecticide susceptibility

**Figure 2:**
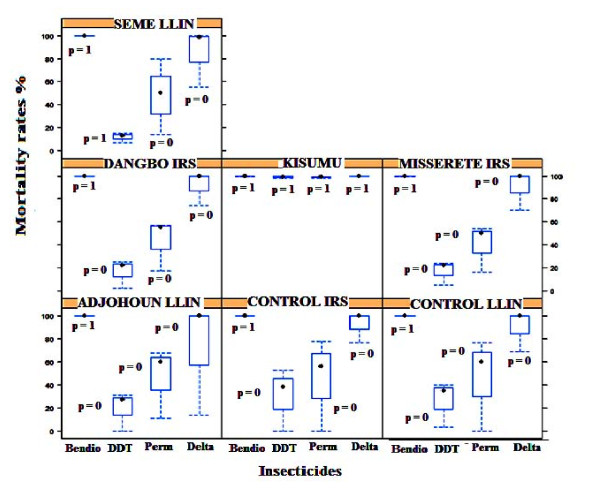
**Variation of mortality rates per insecticide from 2007 to 2010 in each locality**.

The susceptibility of adult mosquitoes (reared from larval collection) to permethrin (0.75%), deltamethrin (0.05%), DDT (4%) and bendiocarb (0.1%) from 2007-10 is presented in Figure 2. The resistance status of the mosquitoes was based on the decrease in the mortality rates according to WHO criteria. From 2007-10, the susceptible strain Kisumu of *An. gambiae *displayed mortality rates above 98% for the 4 insecticides tested (Figure 2). The 24 h post-exposure mortality rate of *An. gambiae s.l *from all localities showed resistance to DDT, permethrin and deltamethrin. In contrast, these mosquitoes were highly susceptible to bendiocarb with a mortality rate more than 99% (Figure 2). Univariate logistic regression, performed with mortality rate as the dependent variable and localities as a covariate with ANOVA test, showed that the phenotypic resistance to DDT and pyrethroid was associated with the localities (p < 0.05). Indeed, logistic regression performed, showed a decrease of mosquito

susceptibility for permethrin (OR = 0.70 [0.51 - 0.95]), p < 0.05), deltamethrin (OR = 0.27 [0.15 - 0.51]), p < 0.05) and DDT (OR = 0.16 [0.11-0.24]), p < 0.05) in Seme LLIN and for DDT (OR = 0.54 [0.39 - 0.75]), p < 0.05) in Adjohoun LLIN. This finding was similar to Misserete IRS for permethrin (OR = 0.53 [0.39 - 0.72]), p < 0.05), deltamethrin (OR = 0.27 [0.15 - 0.51]), p < 0.05) and DDT (OR = 0.47 [0.34 - 0.66]), p < 0.05), to Dangbo IRS for permethrin (OR = 0.63 [0.47 - 0.84]), p < 0.05) and DDT (OR = 0.51 [0.37 - 0.71]), p < 0.05) compared to the control IRS area. Concerning the mortality rate of *An. gambiae *to deltamethrin (OR = 0.51 [0.26 - 1]) and permethrin (OR = 0.82 [0.6 - 1.12]) in Adjohoun they were similar (p > 0.05) to the control LLIN area. The susceptibility to bendiocarb did not change in the LLINs and IRS areas compared to the control area (p > 0.05).

### *Kdr *and *ace.1^R ^*frequencies in survivors and dead (susceptible) *An. gambiae s.l*. to insecticides

*Ace-1^R ^*mutation was not detected in 2 survivors and all 200 randomly drawn dead mosquitoes from the reared strain of *An. gambiae s.l *specimens which were scored for the allele. The *kdr *genotyping performed on dead and surviving mosquitoes to deltamethrin showed that 100% of them were *An gambiae s.s *M form. During the study period, *kdr *frequencies in alive and dead mosquito specimens from Dangbo IRS, Misserete IRS, Seme LLIN and Control area have been relatively high and has not varied significantly (p > 0.05) (Tables 2, 3). *Kdr *frequencies were respectively in the range of 0.78-0.91 for alive and 0.72-0.90 for dead mosquitoes (Tables 2, 3). Whereas in the Adjohoun LLIN area, it was stable at 0.60-0.64% in 2007-09 and varied to 0.83 in 2010 for live specimens (Table 2). This is the same with the *kdr *allelic frequencies in dead mosquitoes, specimens which were in the range of 0.67-0.66% in 2007-09 and varied to 0.77 in 2010 (Table 3). The correlation coefficients between the *kdr *frequency in survivors and mortality rates to deltamethrin were respectively -0.54 (P > 0. 05), -0.43 (P > 0. 05) and 0.84 (P < 0.05) in 2007 year, 2009 and 2010.

**Table 2:** *Kdr *frequency in surviving *An.gambiae s.l. *population 24 h post-exposure to insecticides

Locality	Years	Number of survivors tested	Species Ag	Molecular forms M	*kdr *mutation			
					
					RR	RS	SS	*kdr *frequency (%)
Control area (Plateau zone)	2007	27	27	27	21	6	0	0.89^a^
	
	2009	30	30	30	24	6	0	0.90^a^
	
	2010	25	25	25	22	3	0	0.86^a^

Control area (Flood zone)	2007	25	25	25	21	4	0	0.92^a^
	
	2009	28	28	28	23	5	0	0.91^a^
	
	2010	25	25	25	22	3	0	0.82^a^

Adjohoun (LLIN area)	2007	50	50	50	22	20	8	0,64^a^
	
	2009	30	30	30	11	14	5	0,60^a^
	
	2010	24	24	24	16	8	0	0.83^b^

Dangbo (IRS area)	2007	58	58	58	37	17	4	0,78^a^
	
	2009	60	60	60	38	22	0	0.82^a^
	
	2010	25	25	25	19	6	0	0.88^a^

Misserete (IRS area)	2007	56	56	56	40	16	0	0.86^a^
	
	2009	30	30	30	23	6	1	0.87^a^
	
	2010	22	22	22	18	4	0	0.91^a^

Seme (LLIN area)	2007	54	54	54	49	1	4	0,92^a^
	
	2009	21	21	21	19	2	0	0,95^a^
	
	2010	25	25	25	20	5	0	0,90^a^

Ag: *An. gambiae*; Numbers in the same column sharing the same superscript do not differ significantly (p > 0, 05)

**Table 3:** *Kdr *frequency in dead *An.gambiae s.l. *population 24 h post-exposure to insecticides

Locality	Years	Number of dead tested	Species Ag	Molecular forms M	*kdr *mutation			
					
					RR	RS	SS	*kdr *frequency (%)
Control area (Plateau zone)	2007	27	27	27	20	7	0	0.87^a^
	
	2009	30	30	30	22	8	0	0.87^a^
	
	2010	25	25	25	20	3	2	0.86^a^

Control area (Flood zone)	2007	25	25	25	20	5	0	0.90^a^
	
	2009	28	28	28	20	8	0	0.86^a^
	
	2010	25	25	25	19	3	3	0.82^a^

Adjohoun (LLIN area)	2007	50	50	50	21	25	4	0.67^a^
	
	2009	30	30	30	15	10	5	0,66^a^
	
	2010	24	24	24	14	9	1	0,77^b^

Dangbo (IRS area)	2007	58	58	58	33	19	6	0,73^a^
	
	2009	60	60	60	34	22	4	0,75^a^
	
	2010	25	25	25	15	6	4	0.72^a^

Misserete (IRS area)	2007	56	56	56	39	17	0	0.85^a^
	
	2009	30	30	30	22	6	3	0.81^a^
	
	2010	22	22	22	18	4	3	0.80^a^

Seme (LLIN area)	2007	54	54	54	45	5	4	0.88^a^
	
	2009	21	21	21	15	5	1	0,83^a^
	
	2010	25	25	25	19	4	2	0,84^a^

Ag: *An. gambiae*; Numbers in the same column sharing the same superscript do not differ significantly (p > 0, 05)

## Discussion

The results have shown that *An. gambiae s.s*. M form was the major malaria vector species biting in the Oueme region. This corroborates previous reports [21] of the anopheline distribution in southeast Benin, which explained the absence of the S molecular form by the ecological characteristics of the Oueme region that did not support its selection. The findings have also shown that *kdr *gene frequencies were stable from 2007 to 2009 in the LLIN area but had significantly increased in 2010. Despite the LLIN distribution, the cause of this stability of *kdr *gene frequencies from 2007 to 2009 is unknown, because the same results were obtained in the control area that has not benefited from the distribution of LLINs. But, a similar trend reported by a study in Bioko between 1998 and 2001 showed no evidence of *kdr *in the *An. gambiae s.s*. population despite the use of pyrethroid-impregnated bednets [36]. It was on the basis of this study that the decision was made to implement IRS with a pyrethroid insecticide in Bioko [37]. However, in 2010, a significant increase of kdr mutation frequency was observed in Dangbo, Misserete, Adjohoun and Seme LLIN around 23 months after the first LLIN distribution, and 16 months after the second. This increase was corroborated with the strong correlation (correlation coefficient R^2 ^= 0.84; P < 0.05) between the *kdr *frequency and the survival rate obtained among the *An. gambiae s.l*. populations tested with deltamethrin. Indeed the main mechanism of resistance to pyrethroids is the mutation Leu 1014F *kdr *allele in Benin. Recent studies have shown that this mutation is expanding in the South [21,38] and North Benin [38]. This high mutation could explain the resistance to deltamethrin in *An. gambiae *collected from HLC and breeding sites of all localities including the control area, in 2010, two years after the implementation of vector control. Similarly, the resistance to permethrin and DDT has been maintained and became higher. These findings corroborate previous studies that had reported resistance of *An. gambiae *to DDT and permethrin in Benin [18,21,39] and in Ethiopia [40] to DDT, permethrin and deltamethrin. Although suspected, the selective pressure exerted by the promotion of mosquito nets by the Ministry of Health and the free distribution of LLINs in the Oueme region, causing the *kdr *increase within *An. gambiae *populations is doubtful. Because the findings showed a significant decrease in deltamethrin mortality rates from 85% to 46% in LLIN area and 32% to 22% in IRS area and control area. Other previous studies have shown that the selection of resistance to pyrethroids in the populations of malaria vectors was due to the extensive use of LLIN [41,42]. Hence, resistance selection in the *An. gambiae *population to deltamethrin seemed most likely to have been developed as a consequence of exposure of adult mosquitoes to this insecticide from LLINs distributed in LLIN areas. Moreover, the high domestic pyrethroid use [18,21], the contamination of soil by using pesticides for crop protection [18] in the Oueme region, could justify the resistance to deltamethrin in control and IRS areas who had not benefited from the distribution of LLINs. This hypothesis was supported by previous studies in Mali that showed an increase in *kdr *frequencies in the absence of any wide-scale control program [12]. This diversity of factors (LLINs, IRS, mosquito coils, aerosols, use of pesticides for crop protection) that select individual resistant *An. gambiae *could also explain the spatial variation of low susceptibility of mosquitoes to insecticides. *Kdr *resistance gene was not the only mechanism of resistance observed in the Oueme region. This could justify the highest *kdr *frequency observed among the strain susceptible to deltamethrin. According to previous studies in Benin, high activity of esterases and oxidases was detected in populations of *An. gambiae *and *Culex quinquefasciatus *resistant to pyrethroids [26]. Hence, further investigations are required to determine the role of *kdr *in conferring resistance and the presence of other resistance mechanisms involved in the different classes of insecticides [43]. Indeed, when exposed to several insecticides, *An. gambiae *develops a resistance to these chemicals through several mechanisms of adaptation. Therefore, major challenges to malaria control in Africa must include the monitoring of resistance of mosquitoes to insecticides, but should also involve the education of people on the appropriate use of insecticides.

After four rounds of bendiocarb IRS from 2008 to 2010, *An. gambiae *remained susceptible to bendiocarb. This finding is in agreement with a previous study conducted in Benin [20,21,44] and in Bioko where the number of *An. gambiae s.s*. exiting through window traps were significantly reduced and remained low with subsequent IRS rounds with a bendiocarb [4]. This susceptibility of *An. gambiae *to bendiocarb may be explained by the absence of individual homozygous RR in the Oueme region. In *Culex pipiens *populations the *ace-1 *mutation has been associated with a high fitness cost [45] and the same may be true in *An. gambiae s.s*. as the frequency of the *ace-1 *mutation in mosquito populations declines rapidly after a few generations in the absence of selection pressure from organophosphates or carbamates insecticides [46]. Similarly, Djogbenou *et al. *reported that the main cost of resistance found for *An. gambiae *mosquitoes homozygous for the G119S mutation was that they were significantly more likely to die during pupation than their susceptible counterparts [47]. But after the fourth round of IRS in 2010 heterozygous (RS) individuals of *ace-1 *mutations were detected in a few localities, with a significant variation (0-13%) of *ace-1 *frequency in Seme IRS in contrast to insignificant increase (p > 0.05) noticed in the localities of Control IRS, Dangbo IRS, Misserete IRS and Dangbo LLIN. This occurrence of heterozygous individuals, in the intervention area in 2010 could be attributed to a high selection pressure, because two rounds of IRS had been carried out that year. However, the occurrence of heterozygous individuals even at sites far from the sprayed areas, suggests other factors than those exerted by the vector control program. The *ace-1 *mutation may have migrated from treated to untreated areas, explaining the parallel increase in those areas. Conversely, the greater frequency of *ace-1 *mutation in *An. gambiae *specimens in Seme IRS, despite the fact that they are interspersed by at least 5 km with untreated control area and LLIN area, may suggest a possible migration of *ace-1 *mutation to untreated areas. If migration is restricted, the selection pressure in the untreated areas may be caused by other than the one induced by IRS. Indeed, recent studies [18,48] showed that this region has a different bioclimatic characteristic with high rainfall (1,500 mm annually), where insecticides are extensively used for agriculture. This suggests that selection of resistant individuals has been caused by insecticides used for other purposes apart from those used by the IRS, although it is difficult to identify the specific activity with the present study.

## Conclusion

The results of this study have confirmed that natural populations of *An.gambiae *in the Oueme region have maintained and developed their resistance to pyrethroids, but are still susceptible to bendiocarb. In Africa the pyrethroid resistance had highly increased in *An gambiae *populations. This increase coincided with the period where chemical vector control was deployed with unprecedented levels of coverage in Africa. In this context there was selection of resistant individuals in the treated areas (LLINs and IRS), however, in untreated or control areas, the selection of resistance recorded may have been caused by unknown factors other than LLINs and IRS. These findings have important implications for malaria vector control programs using IRS and LLIN. Firstly, assessment and monitoring of resistance to pyrethroids and bendiocarb in malaria vector control should be a priority to help correct the current malaria preventive activities and guide in the selection of insecticides to use in the future for malaria vector control in Benin. Secondly, strategies for resistance management [49,50] should be implemented to delay the development or expansion of insecticide resistance by the rotation or the mixture of different classes of insecticides with different target sites. Thirdly, it is necessary to implement a dialogue and partnerships between the fields of health and agriculture in order to coordinate the appropriate use of insecticides with reciprocal benefits for both parties.

## Competing interests

The authors declare that they have no competing interests.

## Authors' contributions

GGP, MS, RO and MA designed the study. GGP, NA and OO carried out the experiments. GGP and FO analyzed the data. GGP and MS drafted the manuscript. MA, GG and MS critically revised the manuscript. All authors read and approved the final manuscript.
